# The fundamental tradeoff in genomes and proteomes of prokaryotes established by the genetic code, codon entropy, and physics of nucleic acids and proteins

**DOI:** 10.1186/s13062-014-0029-2

**Published:** 2014-12-12

**Authors:** Alexander Goncearenco, Igor N Berezovsky

**Affiliations:** Computational Biology Unit and Department of Informatics, University of Bergen, N-5008 Bergen, Norway; Bioinformatics Institute (BII), Agency for Science, Technology and Research (A*STAR), 30 Biopolis Street, #07-01, Matrix, Singapore, 138671 Singapore; Department of Biological Sciences (DBS), National University of Singapore (NUS), 8 Medical Drive, 117597 Singapore, Singapore; Current address: Computational Biology Branch of the National Center for Biotechnology Information in Bethesda, Maryland, USA

**Keywords:** Fundamental tradeoff, Genomes, Proteomes, Prokaryotes, Nucleic acids, Proteins, Structure, Stability, Evolution, Adaptation

## Abstract

**Background:**

Mutations in nucleotide sequences provide a foundation for genetic variability, and selection is the driving force of the evolution and molecular adaptation. Despite considerable progress in the understanding of selective forces and their compositional determinants, the very nature of underlying mutational biases remains unclear.

**Results:**

We explore here a fundamental tradeoff, which analytically describes mutual adjustment of the nucleotide and amino acid compositions and its possible effect on the mutational biases. The tradeoff is determined by the interplay between the genetic code, optimization of the codon entropy, and demands on the structure and stability of nucleic acids and proteins.

**Conclusion:**

The tradeoff is the unifying property of all prokaryotes regardless of the differences in their phylogenies, life styles, and extreme environments. It underlies mutational biases characteristic for genomes with different nucleotide and amino acid compositions, providing foundation for evolution and adaptation.

**Reviewers:**

This article was reviewed by Eugene Koonin, Michael Gromiha, and Alexander Schleiffer.

**Electronic supplementary material:**

The online version of this article (doi:10.1186/s13062-014-0029-2) contains supplementary material, which is available to authorized users.

## Background

While the genetic code inherently bridges the realms of nucleic acids and proteins, causal relations between the nucleotide and amino acid compositions continue to be a topic of intense discussion [1-5]. Degeneracy of the genetic code along with flexibility in the choice of chemically similar amino acids leads to a mutual adjustment of the genomic and proteomic compositions [2,5,6]. Phylogeny and environmental conditions, on the other hand, introduce biases in either or both of these compositions [2,3,5]. Both nucleotide [7-17] and amino acid [1-3,18-29] contents are important determinants of the mechanisms of stability and adaptation [1,4,5,14,18,19,28-33]. Purine load (the (A + G) content [2,5,16,34]) and the (G + C) content [2,8,11,17,28,29,35-37] were shown to be the signatures of thermal adaptation in prokaryotes. Increase of the purine load in coding DNA is to a large extent result of the thermal adaptation of proteins [5], as well as a signal of stabilizing stacking interactions between purine bases in DNA and RNA [2,5,16]. The GC content can be governed by the number of factors, such as genome replication and DNA repair mechanisms [17], involvement into lineage- and niche-specific molecular strategies of adaptation [36], contribution of the codon usage [35] and amino acid composition [11,29,38,39]. Amino acid compositions, in turn, can directly reflect demands on the protein structure and stability [1,3,18-27,33,40-42] and even affect the nucleotide compositions [2,4]. Conversely, protein content can be driven by the nucleotide compositions [11,29,35,38,39]. As a result, causal relationships between the nucleotide and amino acid compositions are very complex, and they depend on various evolutionary and environmental factors [2,4,15,18,19,25,26,29,31,32]. Therefore, the correct and yet unanswered question is how and to what extent the compositions of nucleic acids and proteins affect each other [2]. In order to unravel an intricate connection between them, we considered the realms of natural nucleotide and amino acid compositions and their theoretical limits.

We found that all the genomes are confined within the narrow area along the curve of presumably optimal tradeoff between the compositions of nucleic acids and proteins regardless of the environmental conditions, habitat, phylogeny and other factors. We explored the nonlinear nature of the compositional tradeoff, and we argue that it is governed by the basic properties of the genetic code and can be described analytically. The tradeoff allows predicting amino acid composition in prokaryotes based on the genomic GC with high precision (find prediction of the amino acid composition for the GC content of interest here: http://folk.uib.no/agoncear/GC_AA/). We also simulated random mutations in order to explore the nature and dynamics of the tradeoff. Amino acid depth [43,44] is a parameter that reflects proper compactness and ratio between the hydrophobic core and hydrophilic surface in the native protein globule. We, therefore, used average depth in simulations of mutations as compositional criteria of protein foldability and stability. We show that demand on protein stability is an important if not the major determinant of the tradeoff’s width. The purine/pyrimidine ratio (R/Y) and the GC content were used in the above simulations as compositional determinants of nucleic acids’ stability [2,5,45]. We revealed that in genomes with low GC content the R/Y ratio is increased, and there is an excess of purine-purine (RpR) dinucleotides in both strands of the double-stranded DNA. This dinucleotide bias is directly related to the contribution of purine-purine stacking to stability, pointing to a potential switch from the base paring to base stacking as the dominant mechanism of DNA stability in genomes with low GC content. Despite increased rate of the nonsynonymous mutations in genomes with low GC, we observed persistence of the physical-chemical characteristics in the amino acid substitutions, indicating that both DNA and protein structure stabilizing mechanisms are at play. Overall, we show that in addition to the role of genetic code, the optimization of codon entropy and demands on the DNA, RNA and protein stability are the crucial determinants of the tradeoff. Resulting compositional tradeoff observed here underlies mutational trends and mutual tuning of the nucleotide and amino acid compositions.

## Methods

### Genome database and analysis of compositions, phylogenetic and environmental factors, and analysis of the GC content

**Figure 1:**
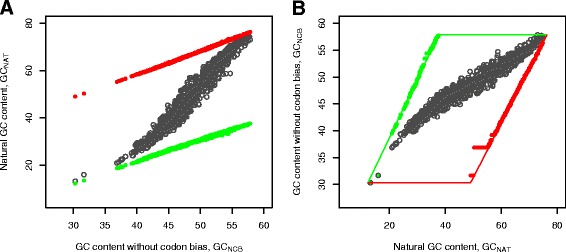
**Distribution of the GC**_**CB**_ **and GC**_**NCB**_ **content and the derived theoretical limits for 1364 prokaryotic genomes. A**, the highest (red) and lowest (green) limits on nucleotide composition obtained by replacing natural codons with synonymous GC-richest and GC-poorest ones. **B**, the theoretical limits (red and green) on amino acid composition (GC_NCB_), see also Additional file 1: Figure S1.

We downloaded 1364 prokaryotic genomes (106 Archaea and 1258 Bacteria, the summary is in Additional file 1: Table S1) from NCBI Genbank and calculated natural GC content (GC_NAT_) of the protein-coding DNA sequences (Figure 1). The average standard deviation of the GC content in individual protein-coding sequences reaches up to 4.5 percent for the genomes with 40 to 65 percent genomic GC and stays within 3.8 percent for other genomes (Additional file 1: Figure S1). The average genomic GC content was used as the characteristic of the genomic nucleotide composition. The GC load of individual amino acids, obtained as the average over the synonymous codons for corresponding amino acid, was used to express the amino acid composition of a proteome in GC units. The GC content of protein-coding DNA without codon bias (GC_NCB_) mimics a random choice of codons. It is calculated as a product of the genomic amino acid frequencies and corresponding GC saturation values, i.e. the average GC content of the amino acid’s codons (Additional file 1: Table S2). We also obtained the GC_max_ and GC_min_ content values by taking the GC-richest and GC-poorest codon for each amino acid, respectively. Prokaryotic genomes exploit a wide range of nucleotide compositions, with the GC content varying from 17 to 76 percent in 1364 genomes analyzed in this work. There is a wide range of theoretically possible combinations of the nucleotide and amino acid compositions. Noteworthy, significant compositional differences were observed for species that are proximal in phylogeny and/or thrive under the same extreme conditions. We considered the following environmental and genomic factors: salinity, optimal growth temperature, oxygen tolerance, domain of life, and habitat. All the factors were assigned according to NCBI Genbank annotations.

We used dinucleotide contrast *C*_*N*1*pN*2_ = *f*_*N*1*pN*2_/(*f*_*N*1_ × *f*_*N*2_) to analyze dinucleotide frequencies and their GC content dependencies. Here, the *f*_*N*1*pN*2_ is an observed frequency of the dinucleotide N_1_pN_2_, and *f*_*N*1_ and *f*_*N*2_ are natural frequencies of the nucleotides N_1_ and N_2_.

We used average amino acid depth [43,44] as a parameter that reflects proper compactness and ratio between the hydrophobic core and hydrophilic surface in the native protein globule. Since it can be deduced purely from the amino acid compositions, we calculated a proteomic average of the amino acid depths. For 1364 prokaryotes under study, the proteomic depth persists in a very narrow interval (0.96-1.02) throughout the whole range of the genomic GC.

### Nonlinear least squares regression

**Figure 2:**
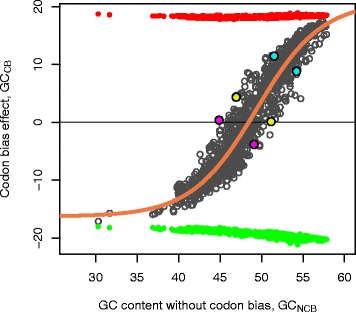
**The tradeoff between nucleotide and amino acid compositions.** Theoretical model. Black circles represent the genomes. The lower (green) and upper (red) limits for GC_CB_ are calculated in the same way as in Figure 1a. The tradeoff describes the relation between the two components of GC depicted by an orange curve (with the coefficients *a* = 20.82, *b* = −16.28, *c* = −49.4, *r* = 0.255). The colored circles illustrate three pairs of genomes with the same GC_NAT_ (45 percent – magenta, 51 – yellow, and 63 – blue) obtained by combining the GC_CB_ and GC_NCB_ in different proportions (see Additional file 1: Table S7 for details).

We used constrained weighted nonlinear least squares (R software, nls routine, “port” algorithm [46]) to fit parameters of the logistic function *GC*_*CB*_(*GC*_*NCB*_) (Figure 2). The theoretical limits showed in Figure 1 were used as min/max constraints. Because the genomes are not distributed evenly in the range of GC_NAT_ we assigned weights $w\kern0.5em =\kern0.5em - \ln \left(pdf\left(G{C}_{NAT}\kern0.5em -\kern0.5em \overline{G{C}_{NAT}}\right)\right)$, where $\overline{G{C}_{NAT}}$ is the average across all the genomes. The *pdf* is a probability density function of the GC content being different from the average, which is estimated by fitting a mixture of Gaussian distributions (Additional file 1: Figure S2).

### The GC saturation scale and standardized (z-scored) amino acid frequencies

The amino acid component of natural GC content, GC_NCB_, is a manifestation of the cumulative contribution from all 20 amino acids. To quantify a relationship between the nucleotide and amino acid compositions directly, we introduced the “GC saturation scale” (Additional file 1: Figure S3, Additional file 1: Table S2). The scale shows an average percentage of guanine and cytosine bases in the codons of each amino acid (Additional file 1: Figure S3). There are three groups of amino acids according to their GC saturation: GC-rich (PGARW), GC-medium (MLCDEHQSTV), and GC-poor (IPKNY).

We standardized frequencies *f* of the amino acids belonging to the same GC saturation group *P*, and calculated combined z-scores for each GC saturation group in each genome ${Z}_P\kern0.5em =\kern0.5em \frac{1}{n}{\displaystyle \sum_{i\in P}\frac{f_i\kern0.5em -\kern0.5em {\overline{f}}_l}{\sigma_i}}$, where ${\overline{f}}_i$ is the average frequency of the amino acid *i* in all the genomes, *n* is the number of amino acids in the group *P*, and *σ*_*i*_ is the standard deviation. The standardized fraction of amino acids (z-score) with medium GC saturation shows almost no correlation with the GC_NCB_ (Pearson’s r = 0.29, Additional file 1: Figure S3). The z-scores of amino acids with low GC saturation are strongly anti-correlated with GC_NCB_ (r = −0.99), whereas z-scores of highly GC-saturated amino acids are strongly correlated with GC_NCB_ (r = 0.98, Additional file 1: Figure S3). Thus, frequencies of amino acids at the extremes of the GC saturation scale change at the expense of each other.

### Amino acid content prediction based on genomic GC content

As shown in the previous section GC_NCB_ represents the amino acid composition, thus allowing one to predict an amino acid content given the genomic GC. Prediction is a two-step procedure: first, the GC_NCB_ value is obtained from the genomic GC; second, amino acid content is derived from GC_NCB_. Genomic GC is a combination of the average (non-codon-biased) GC load of amino acids (GC_NCB_), codon bias effect (GC_CB_), and a contribution from the RNA-coding genes and intergenic regions. In prokaryotes, the GC content of protein-coding DNA determines genomic GC [2]. Therefore, it is safe to neglect contribution from the RNA-coding genes and intergenic regions without any significant loss in the prediction’s precision (Additional file 1: Table S3). Using the compositional tradeoff model for the codon bias (*GC*_*CB*_) as a function of the non-codon biased GC content (*GC*_*NCB*_), an optimal combination of GC_NCB_ and GC_CB_ given the genomic GC can be found in the optimization procedure with the target (*GC* − *GC*_*NCB*_ − *GC*_*CB*_(*GC*_*NCB*_))^2^ → *min*. Once the GC_NCB_ value is found, the amino acid frequency *f* can be predicted as: *f* = (*αGC*_*NCB*_ + *β*)*σ* + *μ*, where *μ* is the mean value and *σ* is the standard deviation of the amino acid frequency taken from Additional file 1: Table S2. The parameters *α* and *β* can be found for each amino acid individually, but it is also possible to take advantage of the grouping arrangement of amino acids according to their GC saturation, thereby decreasing the total number of fitted parameters in the predictor. For GC-poor amino acids *α* = −0.213 and *β* = 10.334, while for GC-rich amino acids *α* = 0.211 and *β* = −10.232 (Additional file 1: Figure S3). In GC-medium group, where the correlation between standardized amino frequencies and GC_NCB_ is low, we took the average values of natural frequencies in all prokaryotes. However, for valine, serine, and histidine belonging to the GC-medium group, the individual linear regression models can be used to improve the prediction performance up to R^2^ = 0.51, 0.37, and 0.22, respectively (Additional file 1: Tables S3, S4). The web-based predictor of the amino acid compositions (http://folk.uib.no/agoncear/GC_AA/) calculates amino acid frequencies using the tradeoff model (described in Results section) and individual linear regressions for each residue type.

The accuracy of amino acid composition’s prediction relies on the correctly determined GC_NCB_ values. We use chi-squared test to assess how well the logistic model fits the data. We split the range of natural and predicted GC_NCB_ values into *k* intervals. For *k* = 11, number of degrees of freedom is equal to 6 (with 4 regression parameters in the tradeoff model), *χ*^2^ = 10.692, p-value = 0.0984. The coefficient of determination (*R*^*2*^) is used to assess the performance of amino acid frequency predictions:

$$ {R}^2\kern0.5em =\kern0.5em 1\kern0.5em -\kern0.5em \frac{{\displaystyle {\sum}_{i=1}^n}{\left({y}_i\kern0.5em -\kern0.5em {\widehat{y}}_i\right)}^2}{{\displaystyle {\sum}_{i=1}^n}{\left({y}_i\kern0.5em -\kern0.5em y\right)}^2} $$

where *y* is natural amino acid frequency, *ŷ* is a predicted frequency of corresponding amino acid, $\overline{y}$ is average frequency of corresponding amino acid frequency in all genomes (see Additional file 1: Table S2), and *n* is the number of genomes.

The root mean square error (RMSE) measures the accuracy of the amino acid frequency predictions: $RMSE\kern0.5em =\kern0.5em \sqrt{\frac{1}{n}{\displaystyle \sum_{i=1}^n}{\left({y}_i\kern0.5em -\kern0.5em {\widehat{y}}_i\right)}^2}$, which should be less than the standard deviation of the observed values. Additional file 1: Table S3 contains *R*^*2*^ and RMSE measurements for the whole set of genomes.

### Simulations of random mutations in relation to the tradeoff

We simulated random mutations by using a compositional substitution matrix based on the nucleotide frequencies of the original (wild type) genome [47]. The goal here was to survey changes in the codon composition caused by mutations given the genomic nucleotide composition. It is important to keep the nucleotide composition unchanged in order to explore composition-dependent trends. Otherwise, the affinity to change composition will dominate the simulation process. As an illustration, we simulated mutations with unnatural substitution matrix where the bases are equiprobable (1/4 each). All the simulation traces converged to one point, corroborating importance of preserving the original composition (Additional file 1: Figure S4). We fixed the nucleotide content by using compositional substitution matrix of the original genome and allowing the codon and amino acid compositions to change freely without any selection applied. A compositional mutation is simulated as follows. First, we choose a codon to be mutated with the probability proportional to its genomic frequency. Second, we randomly (with uniform probability) choose one of the positions in the codon. The selected nucleotide is then mutated according to probabilities in the nucleotide substitution matrix [47]. Codon frequencies are updated as a result of mutations, while the substitution matrix is kept unchanged.

**Figure 3:**
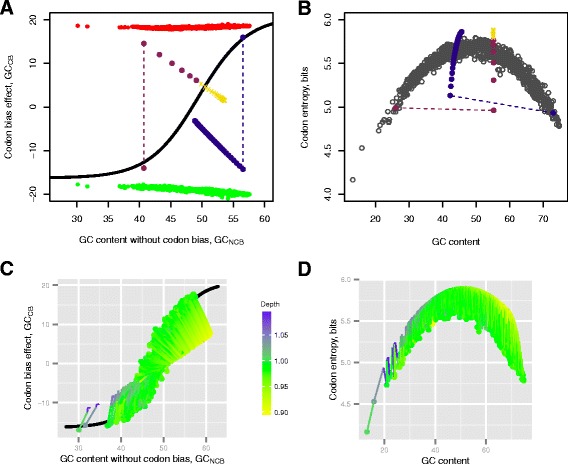
**Simulations of the tradeoff dynamics via random nucleotide mutations. A**, **B**, simulations for two genomes (*Nocardiopsis dassonvillei subs. dassonvillei DSM 43111*, GC_NAT_ = 72.7; and *Streptobacillus moniliformis DSM 12112*, GC_NAT_ = 26.3) with distorted codon bias. **A**, changes in the GC_CB_/GC_NCB_ pairs related to the tradeoff. **B**, the increase of the genomes’ codon entropy in relation to the distribution of entropies in all genomes. **C** and **D**, simulations of mutations in 1364 natural genomes; the simulation starts at points marked by filled circles and continues along the lines. The traces are colored by average amino acid depth **C**, changes in the GC_CB_/GC_NCB_ pairs related to the tradeoff. **D**, behavior of the codon entropy.

In the first experiment, we simulated dynamics of the nucleotide/amino acid content in genomes with strongly distorted (from natural) codon bias. Using constrained optimization by linear approximation method implemented in SciPy (http://www.scipy.org/) we substantially changed the codon bias in *Streptobacillus moniliformis DSM 12112* and *Nocardiopsis dassonvillei subs. dassonvillei DSM 43111* to desired value, preserving, however, their amino acid composition and the GC_NAT_ content. We used the genomes with distorted codon bias as the starting points in the simulations, allowing the nucleotide and amino acid compositions to change freely. We made 2⋅10^7^ mutations (Figure 3A, B) in simulations of each genome, calculating the following characteristics every 10 000 mutations: codon entropy, GC_NAT_, GC_CB_, GC_NCB_, nucleotide composition and its purine/pyrimidine ratio, the number of synonymous and nonsynonymous substitutions, amino acid composition, and the average amino acid depth index. The points in the plots (Figure 3A, B and Additional file 1: Figure S5) show changes in corresponding characteristics for each step in the simulations.

In the experiments on all 1364 genomes the natural nucleotide compositions and codon biases were used, and both nucleotide and amino acid compositions were allowed to change. The simulations were performed by applying 2⋅10^6^ mutations (simulation traces are shown in Figure 3C, D and Additional file 1: Figure S6a, b). Since it may be hard to trace individual genomes in a combined plot, we show simulations for six representative genomes sampled at different GC values (Additional file 1: Table S5 and Additional file 1: Figure S7).

## Results and discussion

The GC content of protein-coding DNA (GC_NAT_) in 1364 analyzed prokaryotic genomes spans from 13 to 75 percent (Figure 1A, black dots). The GC content with eliminated codon bias (GC_NCB_) represents the GC-load of the amino acid composition (Figure 1B and Additional file 1: Figure S8). The maximal (red dots) and minimal (green) theoretical limits of the GC_NAT_ content are obtained by replacing natural codons with the GC-richest and GC-poorest synonymous codons (Additional file 1: Table S2). These limits indicate that given a natural amino acid composition it is possible to obtain a wide range of GC values provided by the codon bias. The boundaries of the GC content determine, in turn, theoretically maximal and minimal values of the non-codon-biased GC content (GC_NCB_). Noteworthy, a relation between the nucleotide (represented here via GC_NAT_) and amino acid compositions (expressed via GC_NCB_) is asymmetric. The range of allowed GC_NAT_ values is about 40 percent, and the whole range shifts to higher values as GC_NCB_ increases (Figure 1A). The inverse is very different with the maximal interval of GC_NCB_ values about 30 percent (for the values of GC_NAT_ between 35 and 50 percent), which is gradually diminishing at the extremes of the GC scale (Figure 1B).

### The tradeoff between the nucleotide and amino acid compositions

Natural protein-coding GC content (GC_NAT_) content can be represented as a sum of the non-codon-biased GC content (GC_NCB_) and the codon bias (GC_CB_). The nature of the relation between two components of GC_NAT_ in prokaryotic genomes, GC_CB_ and GC_NCB_, is nonlinear (Figure 2). At the extremes of the GC content interval, the codon usage bias approaches its theoretical limits and a contribution from the amino acid composition to protein-coding GC_NAT_ becomes much more pronounced than in genomes with an average GC content. The tradeoff between the nucleotide and amino acid compositions can be expressed via differential equation

$$ \frac{dG{C}_{CB}}{dG{C}_{NCB}}\kern0.5em =\kern0.5em rG{C}_{CB}\left(1\kern0.5em -\kern0.5em G{C}_{CB}\right) $$

where *r* is the maximal *GC*_*CB*_/*GC*_*NCB*_ rate. The solution of this equation can be written in the form of logistic function

$$ G{C}_{CB}\kern0.5em =\kern0.5em b\kern0.5em +\kern0.5em \frac{a\kern0.5em -\kern0.5em b}{1\kern0.5em +\kern0.5em {e}^{-r\left(G{C}_{NCB}-c\right)}} $$

where *a* and *b* are upper and lower limits of GC_CB_ respectively. The inflection point *c* corresponds to the GC_NCB_ value with the rate *r*. Using weighted nonlinear regression (see Methods) we fit the model parameters to the GC_CB_ and GC_NCB_ values of the natural protein-coding genomic sequences. The resulting model,

$$ G{C}_{CB}\kern0.5em =\kern0.5em \frac{37.1}{1\kern0.5em +\kern0.5em {e}^{-0.255\left(G{C}_{NCB}-49.4\right)}}-16.28 $$

quantitatively describes the tradeoff between the nucleotide and amino acid compositions (orange curve, Figure 2). Since in prokaryotes the content of protein coding sequences (GC_NAT_) determines corresponding genomic GC, the same parameters are also applicable to genomic GC content (including RNA-coding genes and non-coding regions) without any significant loss of precision (Additional file 1: Table S3).

Taking advantage of the fact that fractions of amino acids (anti)correlate with GC_NCB_ (see the corresponding section in Methods and Additional file 1: Figure S3), we have challenged the tradeoff model for prediction of the amino acid composition given the genomic GC content. The first step of the procedure is calculation of the GC_NCB_ and GC_CB_ values using the tradeoff model. The root mean square error (RMSE) in prediction of GC_NCB_ using the tradeoff model is 0.85 percent of GC content. The second step is prediction of the amino acid frequencies based on the regression models for the GC-poor/-medium/-rich amino acid groups (see Methods and Additional file 1: Figure S3). The resulting error (RMSE) of predicted amino acid frequencies compared to the natural ones is between 0.2 and 0.91 percent of amino acid content (Additional file 1: Table S3). Predictive power of the tradeoff was additionally tested by determining the amino acid compositions of three recently sequenced genomes (not present in the original set of 1364, Additional file 1: Table S6). For illustration purposes we also provide the web application that predicts amino acid compositions of proteomes given their genomic GC content: http://folk.uib.no/agoncear/GC_AA/.

### Versatility of the tradeoff: phylogeny, life styles, and extreme environments

**Figure 4:**
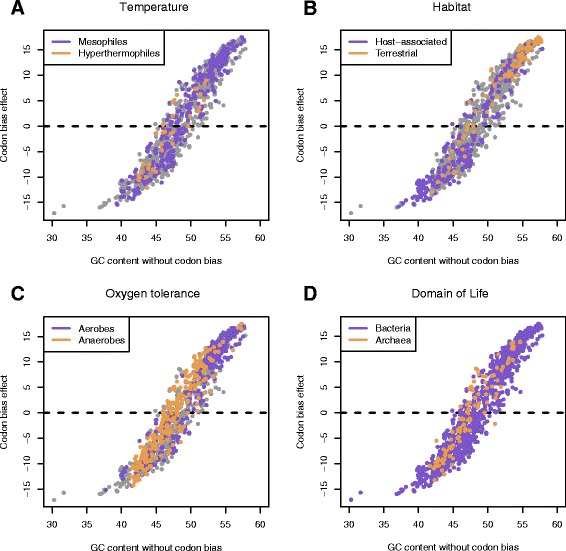
**Environmental factors in relation to the tradeoff: A, Temperature; B, Habitat; C, Aerobicity; D, Domain of Life.**

There are many peculiar nucleotide and amino acid compositional biases, which reflect molecular adaptation to different life styles and environments [2-5,19,26]. We analyzed how different types of genomes are distributed with respect to the tradeoff (Figure 4). Noteworthy, the narrow width of the distribution of genomes around the tradeoff curve (about ±5 percent GC at its maximum, Figures 2 and 4) is sufficient for supporting genomic diversity in archaeal and bacterial domains of life, different life styles, and adaptation to different environments. Adaptation to the same extreme conditions can be achieved via nucleotide/amino acid content pairs located far from each other along the tradeoff’s GC scale (Figure 4). Hyperthermophiles yield the narrowest range of GC values (shown in comparison to mesophiles in Figure 4A) compared to other genomic and environmental factors (Figure 4B-D). Low values of the GC content are typical for host-associated organisms (parasites and symbionts). Terrestrial organisms have higher GC content (Figure 4B), implying that their nucleotide and amino acid compositions are biased in different ways. The GC range in aerobes is wider than in anaerobes, showing an important role of the codon bias in tuning nucleotide compositions of anaerobic organisms (Figure 4C). Archaea has a relatively narrow range of GC compared to Bacteria (Figure 4D), which points to stronger amino acid adjustment in the adaptation mechanisms of Bacteria. The qualitative similarity between Archaea/Bacteria and hyperthermophiles/mesophiles is presumably a consequence of the archeal domination in the hyperthermophilic environments (Figure 4A, B). Regardless of the environmental and lifestyle factors all prokaryotic genomes obey the same tradeoff model, and the RMSE in prediction of GC_NCB_ is less than one percent of GC when the model is applied to a specific subgroup of genomes. The corresponding RMSE values for the subgroups of genomes are: 0.83 – for aerobes; 0.93 – anaerobes; 0.98 – hyperthermophiles; 0.82 – mesophiles; 0.87 – host-associated; 0.63 – terrestrial; 0.94 – Archaea; 0.84 – Bacteria. The most deviating subgroups include hyperthermophiles, Archaea, and anaerobes, likely represented by the same genomes as these groups overlap significantly.

### Determinants of the tradeoff

**Figure 5:**
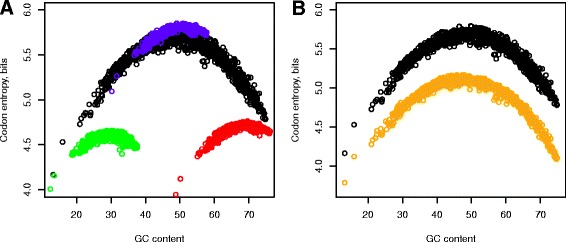
**Limits of the codon entropy in genomes with the natural amino acid composition preserved. A**, Natural nucleotide composition (black); synonymous codons substituted with the GC-richest ones (red) and the GC-poorest ones (green); synonymous codons were used uniformly (blue), GC_NCB_. **B**, Natural nucleotide composition (black); reduced synonymous codons (orange) – in cases when there are several synonymous codons with the same GC saturation, only one codon was used.

**Figure 6:**
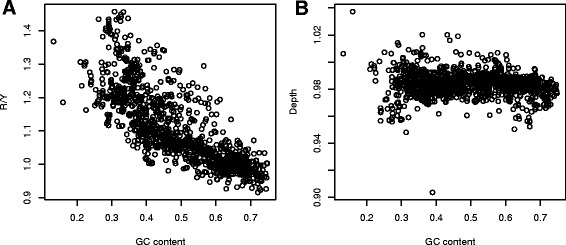
**Dependence of the purine/pyrimidine ratio (A) and the average amino acid depth (B) on the GC content of protein-coding sequences.**

**Figure 7:**
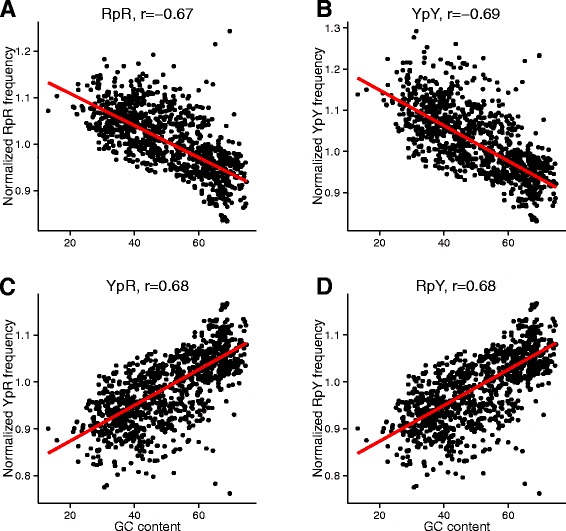
**Dependence of the purine-purine RpR (A) pyrimidine-pyrimidine YpY (B), pyrimidine-purine YpR (C), purine-pyrimidine RpY (D) dinucleotides on the genomic GC**_**NAT**_ **.**

**Figure 8:**
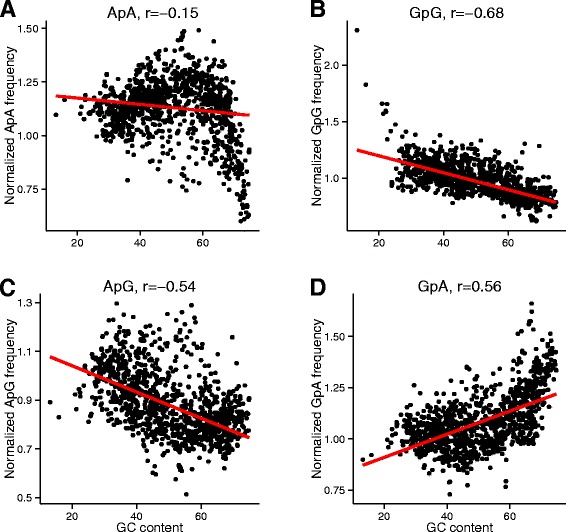
**Dependence of the adenine-adenine ApA (A), guanine-guanine GpG (B), adenine-guanine ApG (C), guanine-adenine GpA (D) dinucleotides on the genomic GC**_**NAT**_ **.**

What are the factors that determine shape of the tradeoff, and why do genomes follow the tradeoff’s curve so closely? First, we explore how the very genetic code sets limits on the compositions of genomes and proteomes. We started from the analysis of Shannon codon entropy (*H* = − Σ *p log*_2_*p*, where *p* is a genomic codon frequency) behavior, in order to understand to what extent it determines a mutual adjustment of the nucleotide and amino acid compositions. The uniform usage of all 61 sense codons gives the absolute theoretical maximum of the codon entropy – 5.93. Codon entropies of natural compositions form an umbrella-like distribution (black dots, Figure 5A, B) with the maximum in the middle of the genomic GC content interval. We further explored the theoretical boundaries of the tradeoff’s entropies by preserving the amino acid composition and changing the codon bias. The GC_NCB_ with uniformly used synonymous codons (blue) represents the upper boundary of the entropy given a particular amino acid composition (Figure 5A). The red and green points show the lower theoretical boundaries of the codon entropy obtained by replacing synonymous codons with the GC-richest and the GC-poorest ones, respectively (Figure 5A). The GC content can also be affected by swapping synonymous codons. Therefore, another theoretical limit for the given nucleotide and amino acid content can be obtained by removing degeneracy in synonymous codons with the same GC saturation. Orange points in Figure 5B show that this boundary is about 0.6 bits lower than the entropies of corresponding natural compositions over the entire range of the genomic GC content. Overall, theoretical limits of the codon entropy show that there is a natural tendency for maximizing codon entropy given the genomic GC content (Figure 5), which is driven by the nature of random mutations and is supported by the redundancy of the genetic code. At the same time, codon entropy does not reach its theoretical maximum given the amino acid content (blue dots, Figure 5A), which points to the existence of additional factors that affect the codon entropies and corresponding nucleotide compositions. Specifically, we found that decrease of the genomic GC content is accompanied by the increase of the purine (A + G) load in the sense strand of the DNA (Figure 6A). A plausible explanation is an existence of the strong contribution from purine-purine dinucleotides to the stability of double-stranded DNA via the base stacking mechanism [2,5,48,49]. Base stacking along with base pairing are two mechanisms that secure stability of the double-stranded DNA [45,49,50]. While GC pairing provides stronger interactions (three hydrogen bonds) than AT pairing (two hydrogen bonds, [45,50]), the purine-purine (RpR) stacking (for all possible dinucleotide combinations of A and G) has lower energy than stacking of other dinucleotides [48,50]. Correspondingly, we found an enrichment of the DNA’s sense strand with purine-purine dinucleotides (Figure 7A), specifically ApA, ApG, and GpG (Figure 8A-C). We also found an increase of the pyrimidine-pyrimidine dinucleotides in the sense strand (Figure 7B and Additional file 1: Figure S9a-c), indicating an abundance of the complementary purine-purine dinucleotides in the anti-sense strand. Thus we conclude that in addition to base-pairing interactions double-stranded DNA is stabilized by stacking interactions provided by ApA, ApG, and GpG dinucleotides (Figure 8A-C and Additional file 1: Figure S9a-c) scattered in different locations in both sense and anti-sense strands. Overall, increase of the R/Y ratio in conjunction with the dinucleotide biases in genomes with low GC (Figures 6, 7, 8 and Additional file 1: Figure S9) reveals an apparent change in the balance between the G•C base pairing [45,50] and the purine-purine base stacking [48,50]. Base pairing is the major contributor to DNA stability throughout most of the GC range. However, the purine-purine base stacking becomes a very important, if not a dominating factor of stability in genomes with low GC content (Figures 6, 7, and 8). Base stacking can also contribute to the stability of a secondary structure (stems) in m-,t-, rRNA, as well as to the stability of single stranded DNA and RNA molecules [2]. Furthermore, demands on the native protein structures and stability imply restrictions on the amino acid composition, thus becoming one of the factors that keep the genomes within a narrow area along the optimal tradeoff (Figure 2). Stability of proteins [51] requires adherence to the optimal ratio between the interior and exterior of the protein globule [52]. The genome-averaged amino acid depths, a distance between the protein’s atom and the nearest bulky water molecules surrounding the protein [43,44], is a characteristic that describes this ratio. We found that values of the averaged proteomic depth are confined within a narrow interval from 0.96 to 1.02 for all 1364 genomes (Figure 6B).

### Boundaries of the tradeoff and its dynamics

What would happen if unnatural combinations of the nucleotide/amino acid compositions emerge, i.e. if the genome is placed far from the optimal tradeoff? We have chosen two genomes at the extremes of the GC_NAT_ scale, *Streptobacillus moniliformis DSM 12112* (GC_NAT_ = 26.3, plum dots in Figure 3A, B) and *Nocardiopsis dassonvillei subs. dassonvillei DSM 43111* (GC_NAT_ = 72.7, navy blue dots) for the following computational experiment. We strongly distorted their codon biases (around 30 percent absolute change in each case, dashed lines in Figure 3A, B), while preserving natural amino acid compositions. Then we applied series of random DNA mutations with probabilities corresponding to the nucleic acid composition of modified genome (see Methods). As mutations accumulated, the GC_CB_/ GC_NCB_ of the genomes followed the shortest path towards the ratio described by the tradeoff model along the isoline of the GC_NAT_ content (Figure 3A). Simultaneously, the Shannon codon entropy (Figure 3B) increased because of the nature of random mutations and a tendency of the compositions near the tradeoff to have high codon entropy. As a result, distorted genomic compositions have gradually converged to its optimal values described by the tradeoff model (Figure 3A, B and Additional file 1: Figure S5). Further, we explored the dynamics of the relationship between the nucleotide and amino acid content by simulating random mutations in all genomes starting from their natural compositions. In order to explore mutational trends depending on the GC content and starting from the assumption that it is a result of the selection that already took place in natural genomes/proteomes, we used the substitution matrix representing the natural nucleotide composition. The simulations show that proteomic-averaged amino acid depth imposes restrictions on the GC_CB_ and GC_NCB_ values, keeping them close to the curve of the optimal tradeoff and pushing the codon entropy to approach its maximum (Figure 3D and Additional file 1: Figure S5). The amino acid depth in mutated genomes (color coded in Figure 3C) with compositions strongly deviated from the tradeoff curve felt outside the naturally observed range of values (green area in Figure 3C corresponding to 0.95 to 1.02 range in Figure 6B). The purine/pyrimidine ratio (R/Y) exploits the whole range of natural values (~1.0-1.4) at low and middle values of the genomic GC content (Additional file 1: Figure S6).

We also explored the composition-dependent mutational trends of the tradeoff. The trend in the GC dependence of the transitions/transversions ratio mimics the codon entropy change (Additional file 1: Figure S10), with the maximum in the inflection point of the compositional tradeoff (Additional file 1: Figure S11 shows the first derivative of the tradeoff). Thus, transversions (changes of purine to pyrimidine or vice-versa) are more likely to take place if the GC content is biased, resulting in the elevated level of nonsynonymous substitutions that reaches highest values at low GC (Additional file 1: Figure S12). This trend roughly corresponds to the purine-pyrimidine ratio (R/Y) behavior (Figure 6A). Therefore, in the genomes with low GC the purine-pyrimidine balance can be affected by an additional constraint on the codon and amino acid compositions. To this end, we considered possible difference in the effects of nonsynonymous substitutions on the amino acid composition. Specifically, if the amino acid is replaced by a chemically similar one, the nonsynonymous nucleotide substitution can be “neutral” from the point of view of the amino acid’s role in the protein structure and stability. In this case, the effect of mutation will be rather negligible, and structure and stability of the protein will remain intact. Using BLOSUM substitution matrices [53] for quantifying similarity between the amino acids, we calculated a substitution score for all simulated nonsynonymous substitutions (Additional file 1: Figure S13) averaged over the genome. The average BLOSUM score for all amino acid substitutions obtained in simulations (Methods) strongly anti-correlates with the GC content of protein-coding DNA (GC_NAT_), with r = −0.92 and −0.89 for BLOSUM30 and BLOSUM62 matrices, respectively (Additional file 1: Figure S13a, b). Thus, in genomes with low GC content, amino acids are more often replaced (on average) by the amino acids with similar physical-chemical characteristics. As a result, in these genomes switching from base pairing to base stacking as the dominating mechanism in DNA stability can take place without compromising stability and function of the encoded proteins.

One can also ask why are there GC-poor and GC-rich genomes? What are the factors that originate and support strong compositional biases? In general, genomic/proteomic compositions emerge as a direct result of the mutational processes [54] and selection acting on the material generated in mutational process [55]. Recently, strong positive correlation was found between the genomic GC content and strength of the coupling between selection on protein sequences and optimization of codon usage in a broad range of Archaea and Bacteria [56]. Selection alone may not sufficient to change the nucleotide composition and to produce extremes of the GC content observed in prokaryotes. One, therefore, should seek for the strong and persistent mutational biases. Two independent works published back-to-back [57,58] unanimously concluded that mutational trends in Bacteria are universally AT-biased (even in Bacteria with high genomic GC content). It has been concluded that if AT-bias would chiefly govern the genomic nucleotide compositions, the latter would inevitably decline down to about 30 percent in all bacterial genomes. Another conclusion in these two works is that natural selection can determine the rates of fixation of AT → GC and GC → AT mutations. Above observations provide a potential explanation for emergence of the GC-poor genomes leaving us with a question about the origin of the GC-rich extremes. A plausible mechanism proposed recently is that bacterial genomes have different Polymerase III mutator genes that may introduce GC-biased mutations depending on the alpha subunit isoforms [59]. In particular, an error prone DNA repair polymerase with dnaE2 alpha subunit may be driving the mutagenesis process towards high GC content.

## Conclusions

Coexistence and mutual adjustment of the realms of nucleotide and amino acid compositions in prokaryotes are the topics of this work. We asked here the most general question – how and to what extent can the nucleotide and amino acid compositions affect each other? The genetic code and codon entropy predetermine mutual adjustment of nucleotide and amino acid compositions depending on the genomic GC content. Specifically, in the middle of the GC content interval (50 ± 5 percent) redundancy of the genetic code allows tuning of the nucleotide content using only the codon bias and not strongly affecting the amino acid composition. However, in genomes with the GC content closer to the upper and lower extremes, the potential of the codon bias is exhausted. Therefore, tradeoff is maintained at the expense of the amino acid compositions, in particular the amino acids with the GC-poor/-rich codons are preferably utilized. Charged amino acids comprise an interesting example of the link between the compositions. Both negatively charged amino acids, aspartate and glutamate, have medium GC saturation. Therefore, they can not be used for the efficient tuning of the nucleotide composition, neither their amount should be significantly affected by possible changes in the nucleotide composition. On the other hand, positively charged lysine and arginine belong to the GC-poor and GC-rich groups. Thus the choice between the lysine and arginine can change the GC content: arginine can be preferred over the lysine in the genomes with high GC content and vice versa.

The most complex relationship in the context of the tradeoff between the nucleotide and amino acid compositions was found in the case of switching between the dominating mechanisms of DNA stability whilst preserving the structure and stability of corresponding proteins. It has been established in numerous experimental and theoretical works that there are two fundamental interactions that determine stability of the double-stranded DNA: base pairing [45,50] and base stacking [48-50]. While GC pairs in the double helix have stronger base-pairing interactions than AT pairs, purines A and G, yield a lower energy of stacking in the purine-purine dinucleotides compared to all others. We found that the codon bias provides a basis for the increase of purine-purine (RpR) dinucleotides in both strands of DNA molecules in the genomes with low GC content. Purine-purine dinucleotide bias secures thus DNA stability, underlies higher stability of the RNA stems and, to lesser extent, single-stranded DNA and RNA molecules [2,5,48-50]. The higher purine content at the low GC values is accompanied by the increase of the non-synonymous mutations in the amino acid sequences. However, most of these amino acid substitutions do not lead to the change of the amino acid type, preserving their physical-chemical features and not compromising structure and stability of the protein. Overall, the interplay between the genetic code, optimization of the codon entropy, and demands on the structure and stability of nucleic acids and proteins chiefly determine the tradeoff throughout the whole interval of the genomic GC values.

To conclude, the tradeoff is a fundamental concept quantifying the non-linear relationship between the nucleotide and amino acid compositions of prokaryotes and allowing one to predict a proteomic amino acid composition based on a single quantity of the genomic GC content (http://folk.uib.no/agoncear/GC_AA/). The tradeoff is purely compositional phenomenon, linking the realms of nucleic and amino acids in prokaryotes regardless of their life styles, environments, and phylogeny. Versatility and diversity in prokaryotic genomes/proteomes is maintained by the tradeoff, which provides a playground for the work of natural selection towards diversification and adaptation.

## Reviewers’ comments

### Reviewer 1: Eugene Koonin, National Center for Biotechnology Information, NIH, Bethesda, Maryland, United States

As far as I can see, the principal feature of the tradeoff (and the justification for using this term) is that in the mid-range of GC-content nucleotide and amino acid compositions are more or less unlinked (adjustment at synonymous positions is sufficient to account for the GC-content) but at the extremes this is no longer the case and amino acid composition trails the GC-content (e.g. preference for Arg over Lys in GC-rich genomes). As the authors point out, the tradeoff is a purely “compositional” phenomenon which is fundamental in the sense that it equally applies to all genomes regardless of any features of the respective organisms. In other words, this is a purely mathematical, “forced” feature of nucleotide sequence that accordingly is in a sense trivial. I do not mean this in a pejorative way: trivial or not it is useful to carefully describe the connections between GC-content and amino acid composition as the authors do in this paper. The interesting effects emerge at the interface of this compositional tradeoff with selection. The paper presents some such effects in particular the higher purine content in GC-poor genomes that apparently is selected for stabilization of DNA.

To me the most interesting question is: why do extremely GC-rich and extremely GC-poor genomes exist at all? It seems that such extremes should be selected against given the inevitable effect on the amino acid composition as per the tradeoff. What gives? The present paper does not address this question.

#### Authors’ response

Questions why there are GC-poor/-rich genomes and what factors originate and maintain these compositional biases are indeed intriguing ones. In general, genomic/proteomic compositions is a direct result of the mutational processes and selection acting upon the results of mutations [55]. Selection alone may not be sufficient to change the nucleotide composition and to produce extremes of the GC content observed in prokaryotes. One, therefore, should seek for the strong and persistent mutational biases. Two independent works published back-to-back [57,58] unanimously concluded that mutational trends in Bacteria are universally AT-biased (even in Bacteria with high genomic GC content). If these biases chiefly governed the genomic nucleotide compositions, the latter would inevitably decline down to about 30 percent in all bacterial genomes. Another conclusion in these two works is that natural selection can determine the rates of fixation of AT → GC and GC → AT mutations. Above observations provide a potential explanation for emergence of the GC-poor genomes leaving us with a question about the origin of the GC-rich extremes. A plausible mechanism proposed recently is that bacterial genomes have different Polymerase III mutator genes that may introduce GC-biased mutations depending on the alpha subunit isoforms [59]. In particular, an error prone DNA repair polymerase with dnaE2 alpha subunit may be driving the mutagenesis process towards high GC content.

What other traits of genomes and proteomes that can originate extreme nucleotide and amino acid compositions, and how can selection affect the tradeoff between them? Recently, for example, strong positive correlation was found between the genomic GC content and strength of the coupling between selection on protein sequences and optimization of codon usage in a broad range of Archaea and Bacteria [56]. However, we are still left to obtain a complete picture of the relations between mutational biases, natural selection, and factors that determine them. Advances in high-throughput sequencing and proteomics provide a wealth of data, diversity and completeness of which will hopefully allow us to answer all outstanding questions.

We have added above discussion and references to the manuscript.

### Reviewer 2: Michael Gromiha, Indian Institute of Technology (IIT) Madras, Tamil Nadu, India

In this work the authors described a fundamental tradeoff between nucleotide and amino acid compositions using a set of more than 1300 prokaryotic genomes. A nonlinear equation has been set to fit the data and analyzed the possible effects on the mutational biases. They have analyzed various factors and different organisms such as mesophiles and thermophiles bacteria and archaea based on habitat and oxygen tolerance. The work is interesting with the combination of physical basis and statistical analysis. The manuscript is well written and sufficient details are provided:

1. The advantages of using nonlinear fit could be discussed.
2. The significance of coefficients in Figure 2 may be discussed.
3. The comparison of features used in Figure 4 using quantitative measures may be useful.

### Authors’ response

1. The nonlinear fit is crucial for exhaustive description of the relationship between the nucleotide and amino acid compositions. It emphasizes on the difference between the compositional tradeoff in genomes in the middle of the GC content interval and those with biased nucleotide compositions. Indeed, there is a strong pressure on the amino acid compositions in genomes with extremely low/high GC contents, resulting in preferential selection of amino acids with GC-poor/-rich codons respectively. The nonlinear nature of the tradeoff can be explored with an interactive web application: http://folk.uib.no/agoncear/GC_AA/. In particular, at GC values close to 50 percent the tradeoff dGC_CB_/dGC_NCB_ > 2.3, whereas at the extremes where GC > 70% or GC < 30% the tradeoff is completely different: dGC_CB_/dGC_NCB_ < 1.0. In case of the linear fit the tradeoff would be constant, which is not the case as exemplified by the genomes at the extremes. Therefore, using a linear fit it is not possible to predict the codon bias effect correctly for the genomes with biased genomic GC content. In order to illustrate this we fitted a weighted linear model GC_CB_ = 1.889 GC_NCB_ - 90.923. If we apply it, for instance, to *Candidatus Zinderia insecticola CARI* genome with GC of 13.2 (Additional file 1: Table S4) it will predict the GC_NCB_ value of 36, and codon bias effect GC_CB_ = −22.8, while the actual value of GC_NCB_ is 30.3 and the most extreme codon bias effect is −17.1. Of course it will be impossible to predict amino acid composition given this high error of the linear model. For all the genomes, the root mean square error (RMSE) of the linear model will be 0.97 percent GC versus 0.85 for the nonlinear model.
2. The model parameters that we obtained for all the available genomes work well for predicting the codon bias and amino acid compositions when applied to different specific subgroups of genomes (see also the answer to question #3). Although we have not estimated the robustness directly, we assume that the weighting by genome abundance across the GC range (see Additional file 1: Figure S2) removes the possible biases originating from non-uniform experimental sampling of the genomes along the GC scale. For completeness we have also obtained the non-linear model parameters for specific groups of organisms considered in Figure 4 (Additional file 1: Table S8). However, we would like to emphasize on the importance of the analytical expression of the tradeoff and predictive power of the general tradeoff model, which correctly describes a relationship between the realms of the nucleotide and amino acid compositions with high precision (down to 1 percent of composition).
3. In order to quantify the differences between the compositions of organisms classified according to different factors in Figure 4, we measured the RMSE, i.e. the error in predicting the codon bias and non-codon biased GC content (GC_NCB_), given the GC content of coding sequences. For all of the genomes the RMSE is 0.85 percent of GC content. The corresponding RMSE values for the subgroups of genomes are: 0.83 – for aerobes; 0.93- anaerobes; 0.98 – hyperthermophiles; 0.82 – mesophiles; 0.87 – host-associated; 0.63 – terrestrial; 0.94- Archaea; 0.84 – Bacteria. According to RMSE the most deviating factors are hyperthermophilies, anaerobes, and archaeal domain of life, which are in fact highly overlapping. Noteworthy, even for the most deviating subgroups the RMSE is within one percent of GC.
  Corresponding explanations and data were added to the manuscript and to the Additional file 1.

### Reviewer 3: Alexander Schleiffer, Research Institute of Molecular Pathology (IMP), Vienna, Austria

This manuscript describes an interplay between nucleotide and amino acid compositions in prokaryotes. More than 1300 genomes both from Archaea and Bacteria were analyzed for their average genomic GC content, and compared to the GC content of individual codons in proteins. Surprisingly, the genomic and the amino acid composition are far more tightly linked than previously thought, and the authors present an algorithm to predict one from the other. This study opens new questions regarding the biochemical/biophysical constraints that determine this relationship.

## Additional file

Additional file 1:
**Compilation of all supplementary figures and tables.** Complete list of supplementary figures and tables is given in the file.

## Abbreviations

GC content: The GC content of complete DNA sequence
GC_NAT_: The GC content of protein-coding DNA
GC_NCB_: The GC content of protein-coding DNA without codon bias, which mimics a random choice of codons
GC_max_ and GC_min_: Are obtained by taking the GC-richest and GC-poorest codon for each amino acid, respectively
