# Supplementary material for: The fundamental tradeoff in genomes and proteomes of prokaryotes established by the genetic code, codon entropy, and physics of nucleic acids and proteins
Source: Biol Direct. 2014 Dec 12;9:29. doi: 10.1186/s13062-014-0029-2 (PMC4273451; doi:10.1186/s13062-014-0029-2)
Supplement: Additional file 1: — Compilation of all supplementary figures and tables. Complete list of supplementary figures and tables is given in the file. [file 13062_2014_29_MOESM1_ESM.zip › 2012097662141560_add1.pdf]

Additional File 1

The fundamental tradeoff in genomes and proteomes of prokaryotes established by the genetic code, codon entropy, and physics of nucleic acids and proteins

Alexander Goncearenco and Igor N. Berezovsky

Table of Contents:

Legends to Figures 2

Legends to Tables 5

Figures 7

Tables 14

Legends to Supplementary Figures

Figure S1. Standard deviation of GC content in individual protein coding sequences.

Figure S2. Mixture of Gaussians describes the probability density function used as a weighting factor in the regression model.

Figure S3. The GC saturation scale. Amino acids are colored and grouped by their GC saturation values. Correlations of standardized fractions of amino acids (z-score) with the GC_{NCB} in three GC saturation groups (left-to-right): GC-poor, GC-medium, and GC-rich.

Figure S4. Simulation traces with equiprobable composition matrix converge to a single point. The depth (shown as the color of the traces) remains within the natural range.

Figure S5. Changes in codon entropy, R/Y ratio, and "depth" during the simulations of two genomes with distorted codon bias. Upper row (A,B,C): *Nocardiopsis dassonvillei subs. dassonvillei DSM 43111* (GC_{NAT} = 72.7), depth is increasing up to the plateau. Bottom row (D,E,F): *Streptobacillus moniliformis DSM 12112* (GC_{NAT} = 26.3), depth is decreasing down to the plateau.

Figure S6. Simulation traces with indication of changes in Purine/Pyrimidine (R/Y) ratio. The simulation starts at points marked by filled circles and continues along the lines. In the interval of low GC content the R/Y ratio reaches the highest values in the natural interval 0.9 – 1.4. **a,** Changes in the purine/pyrimidine ratio related to the tradeoff's logistic function. **b,** Increasing codon entropy.

Figure S7. Simulation traces of six representative genomes sampling the range of genomic GC values.

The color of the trace indicates whether the amino acid depth is within the naturally observed range. Red circles depict the depth within the natural limits. If depth falls outside the range, the trace is shown as yellow crosses. **a,** Simulation trajectories relative to the tradeoff. **b**, Trajectories relative to the genomic codon entropy values.

Figure S8. Obtaining the limits of amino acid composition represented by GC_{NCB} from the constraints on nucleic acid level (GC_{CB}). Black circles represent the GC_{NCB} and GC_{NAT} values in genomes. The red and green filled circles represent the values for the genomes with the most GC-rich and GC-poor codons, respectively, instead of the natural ones. Filled black points show how the inverse limits are calculated from the red and green borders. From the value of GC_{NAT} at the GC_{max} (red) border we obtain the minimal value of GC_{NCB}. From the value of GC_{NAT} at the GC_{min} (green) border we obtain the maximal value of GC_{NCB}.

Figure S9. Dependence of the thymine-thymine TpT (a) cytosine-cytosine CpC (b), cytosine -thymine CpT (c), c thymine-cytosine TpC (d) dinucleotides on the genomic GC_{NAT}.

Figure S10. The ratio of Transitions/Transversions versus the GC content.

Figure S11. The first derivative of the tradeoff by GC_{NCB}.

Figure S12. The ratio of synonymous/nonsynonymous mutations (simulated) versus the GC content.

Figure S13. Nonsynonymous nucleotide substitutions (simulated) result in amino acid changes: a, average BLOSUM30 score of the amino acid substitutions. **b**, average BLOSUM62 score of the amino acid substitutions.

Legends to Supplementary Tables

Table S1. The summary statistics of all genomes used in the study grouped by environmental factors, lifestyle, and domain of Life.

Table S2. Amino acid GC saturation scale. Amino acids are ordered by increasing GC saturation (proportion of GC-rich codons) from left to right. The codon counts are followed by the statistics of amino acid frequencies in prokaryotic genomes.

Table S3. Assessing the quality of the amino acid content prediction based on genomic GC with different regression models.

Table S4. Examples of amino acid content prediction based on genomic GC values in five genomes representing the whole range of nucleic acid compositions. For each genome the first row is natural composition, the second row is prediction based on weighted regression models for individual amino acids.

Table S5. The changes in parameters (R/Y, Entropy H, Depth) during the simulations in six genomes. The Purine/Pyrimidine ratio decreases in all cases except one, and the depth remains mostly unchanged in genomes with the low codon bias. However, the latter decreases in genomes with highly positive codon bias and increases in those with highly negative one.

Table S6. Amino acid content prediction based on genomic GC values for the genomes not listed in the original dataset. Amino acids are ordered by increasing GC saturation from left to right. Predictions of all 20 amino acids based on regression models for GC-rich and GC-poor groups . For each genome the first row represents the natural values, the second row shows predicted values.

Table S7. The nucleotide compositions of three pairs of genomes shown in Figure 2, including their GC_{NCB} and GC_{CB}.

Table S8. Model parameters obtained for the non-linear fit in different groups of genomes.

Figures

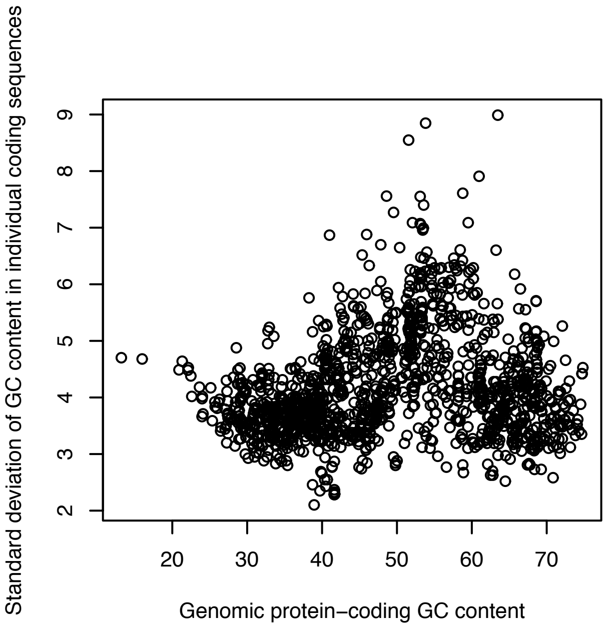

Figure S1

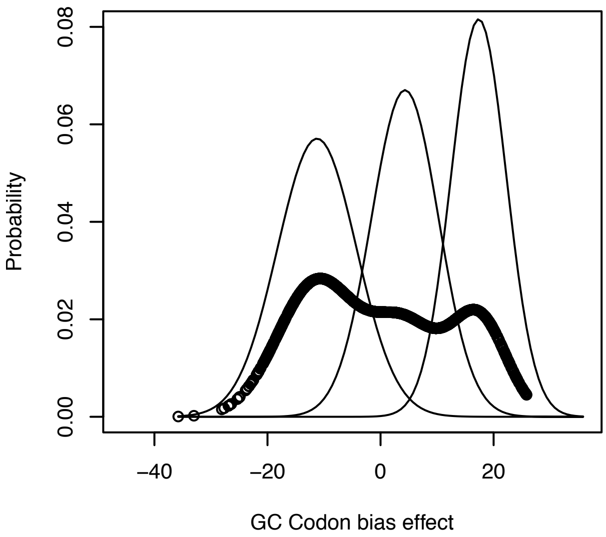

Figure S2

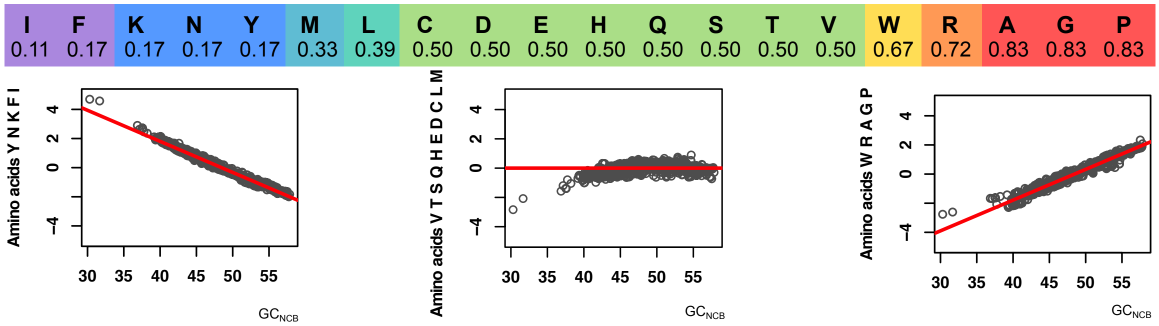

Figure S3

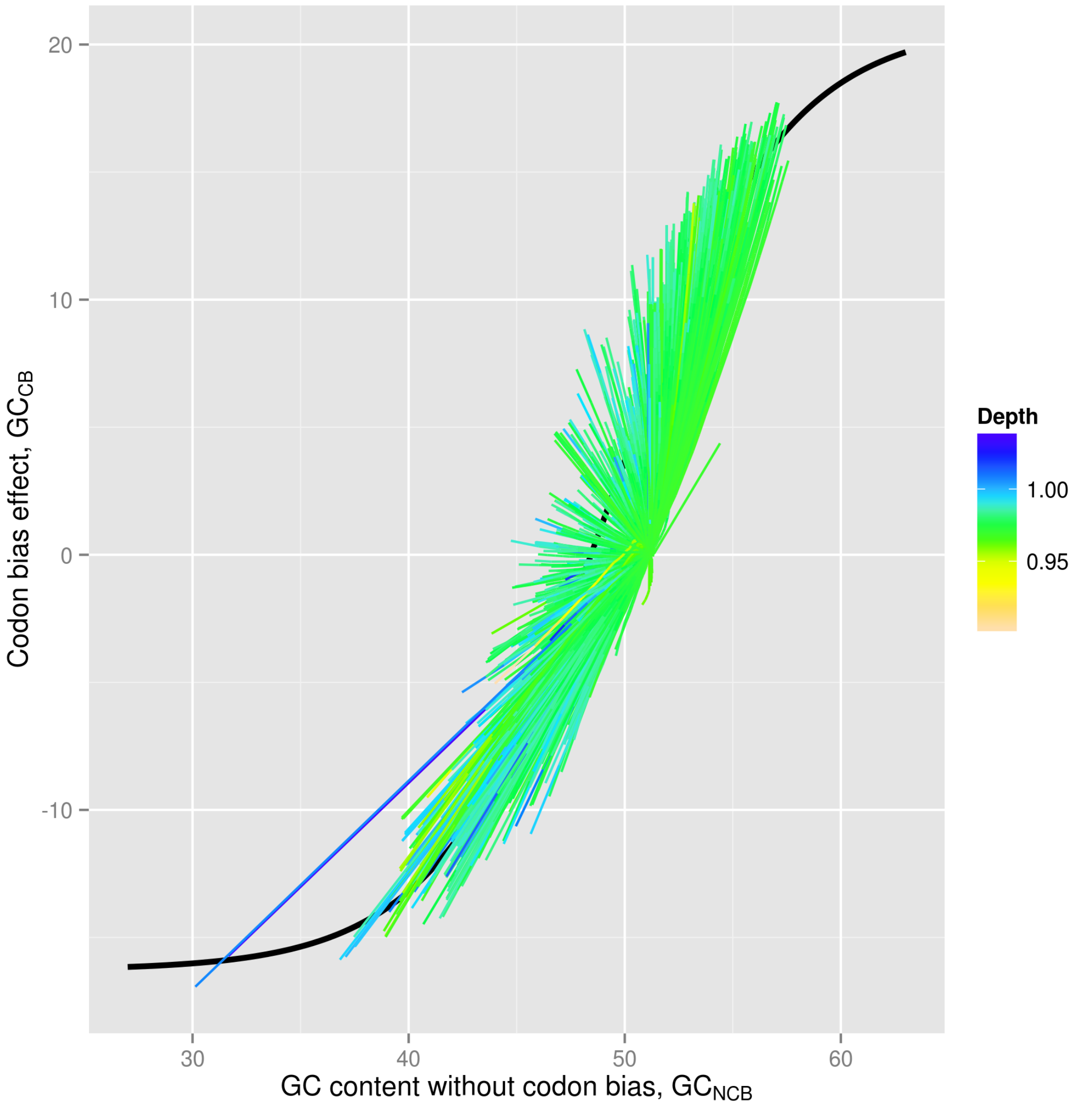

Figure S4

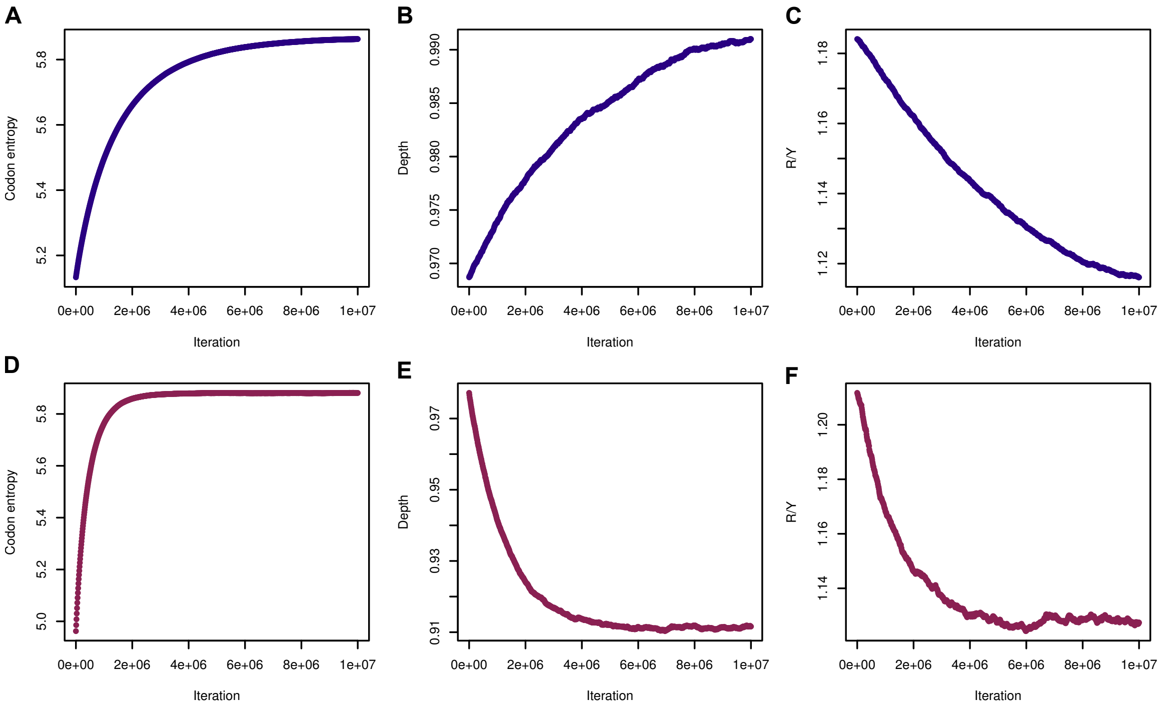

Figure S5

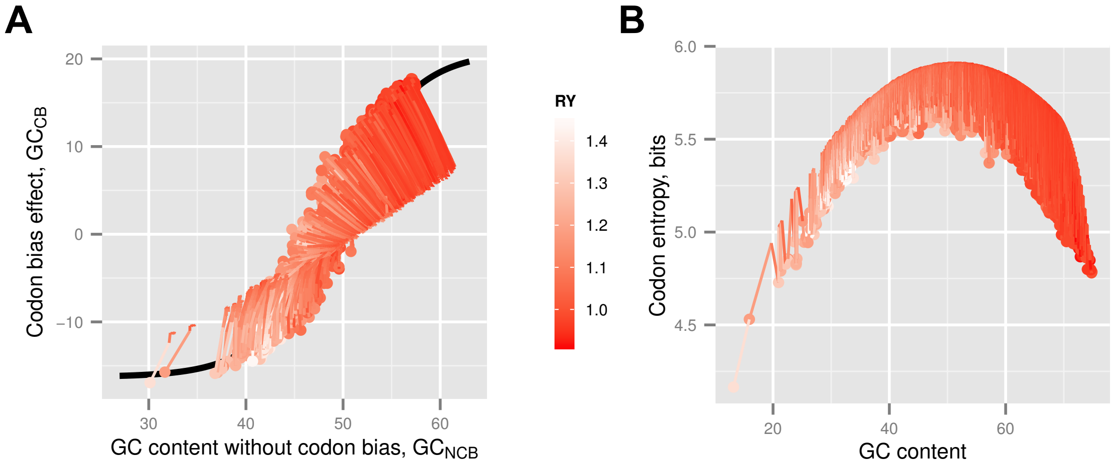

Figure S6

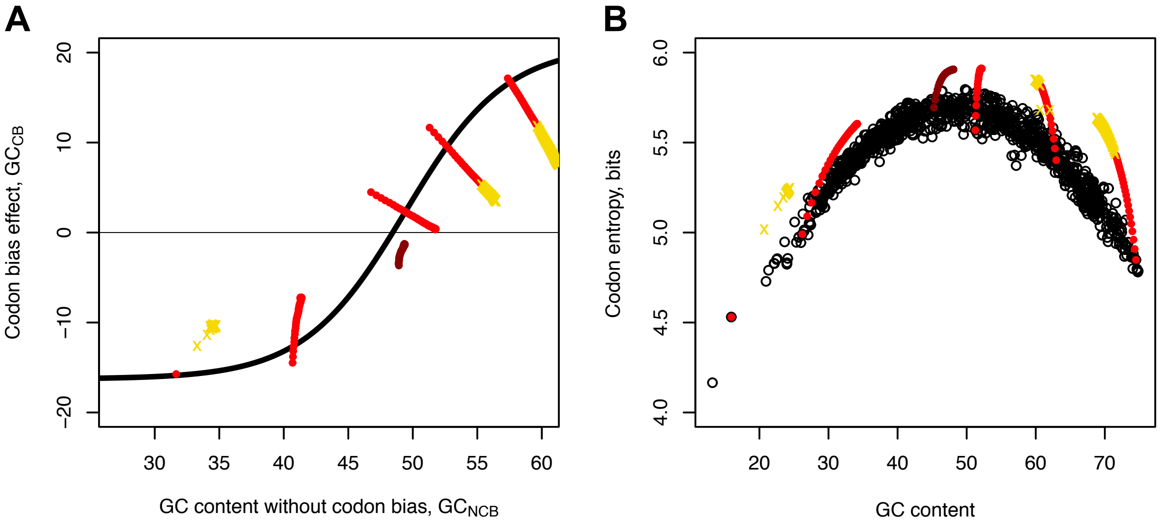

Figure S7

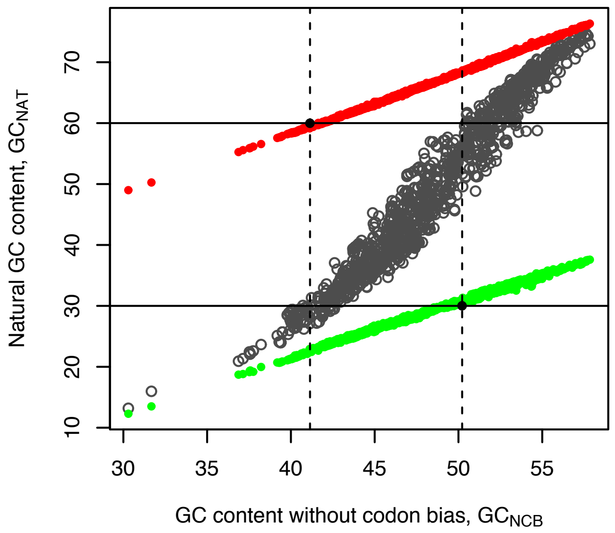

Figure S8

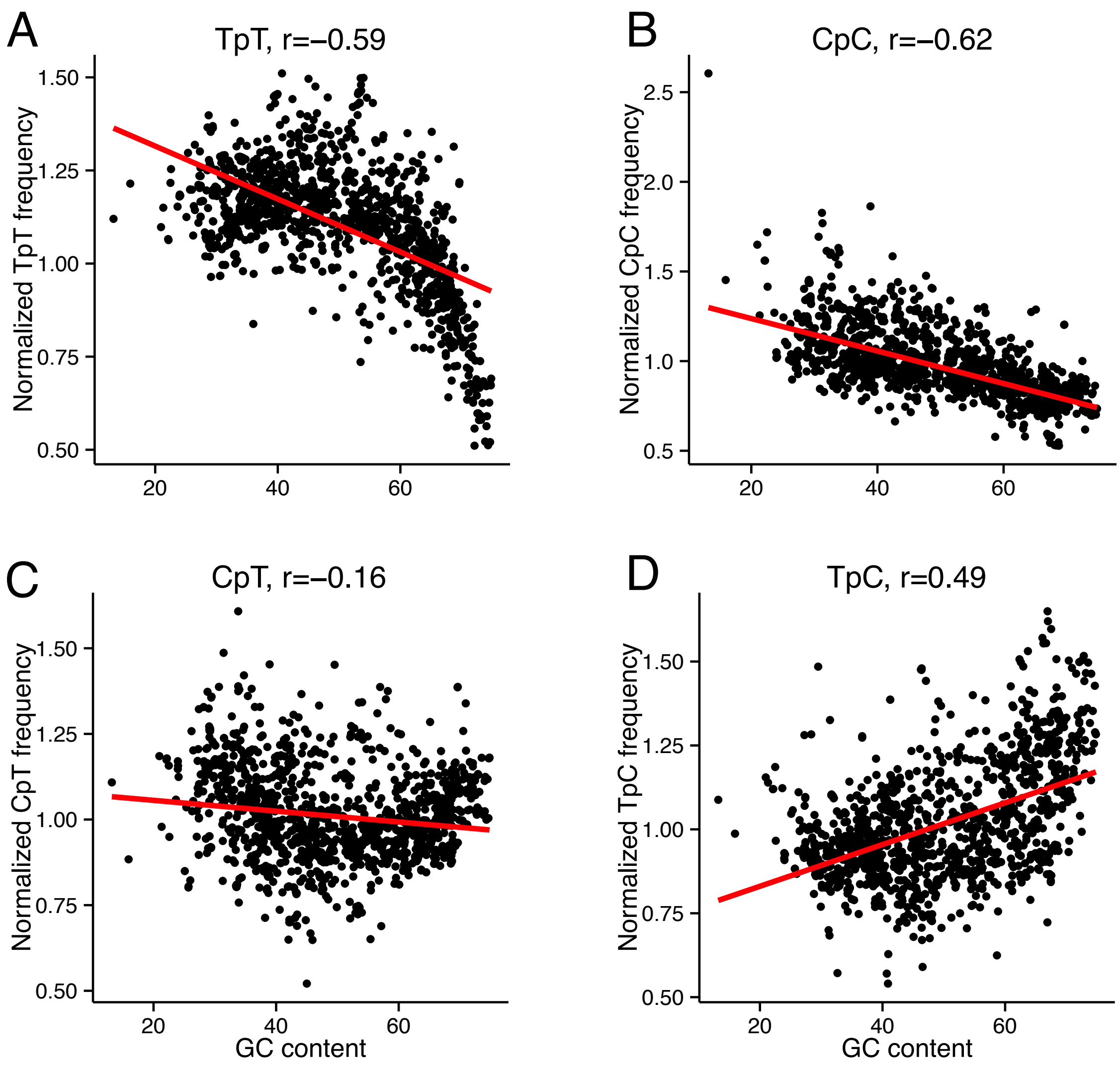
A
TpT, r=−0.59
Normalized TpT frequency
1.50
1.25
1.00
0.75
0.50
20
40
60
B
CpC, r=−0.62
Normalized CpC frequency
2.5
2.0
1.5
1.0
0.5
20
40
60
C
CpT, r=−0.16
Normalized CpT frequency
1.50
1.25
1.00
0.75
0.50
20
40
60
GC content
D
TpC, r=0.49
Normalized TpC frequency
1.50
1.25
1.00
0.75
0.50
20
40
60
GC content

Figure S9

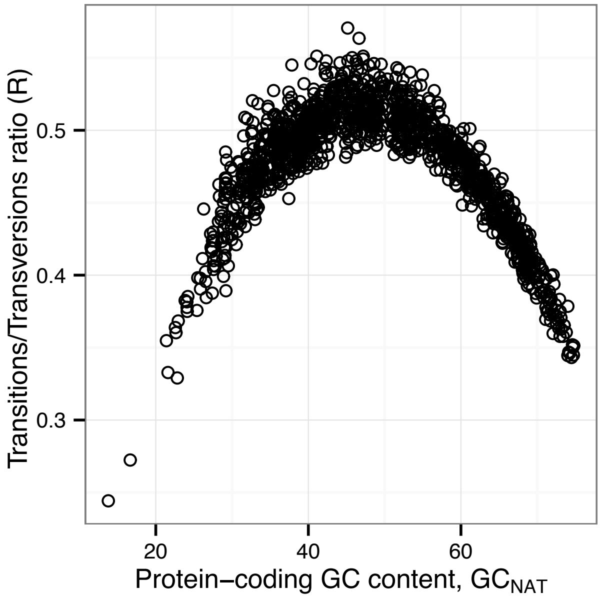

Figure S10

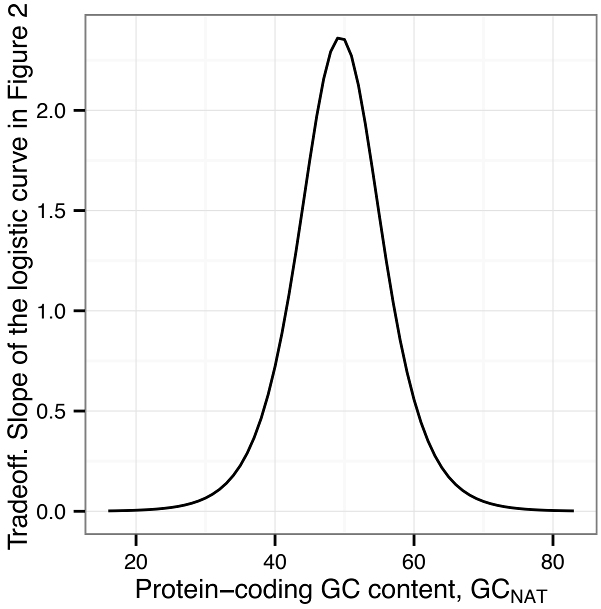

Figure S11

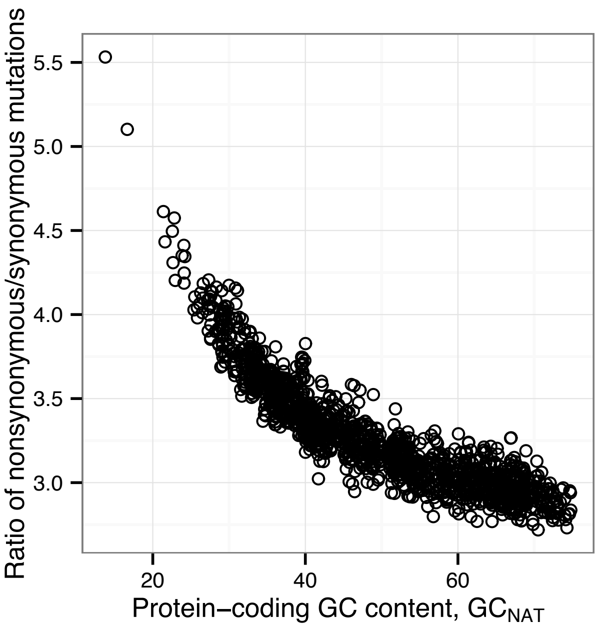

Figure S12

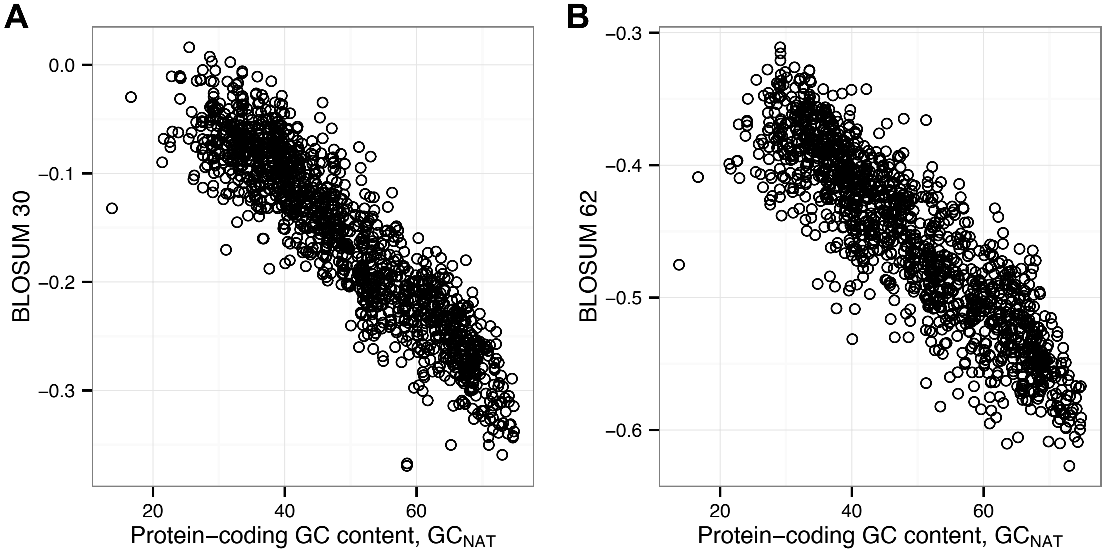

Figure S13

Tables

Genomes total	1364
Genome GC range	16.6 – 74.90
Protein-coding GC range	13.20 – 74.82
Theoretical GC_{min} range	12.28 – 37.58
Theoretical GC_{max} range	49.01 – 76.35
GC_{NCB} range	30.30 – 57.84
Optimal Growth Temperature (OGT) range (˚C)	7 – 106
Psycrophile (OGT < 24 ˚C)	28
Mesophile (24 ˚C <= OGT < 50 ˚C)	394
Thermophile (50 ˚C <= OGT < 80 ˚C)	73
Hyperthermophile (OGT >= 80 ˚C)	40
Host-associated habitat	362
Terrestrial habitat	85
Aquatic habitat	197
Multiple habitat	316
Specialized habitat	144
Aerobic	385
Anaerobic	266
Facultative oxygen	380
Microaerophilic oxygen	36
Extreme halophilic	12
Non-halophilic	168
Mesophilic halophilic	27
Moderate halophilic	29
Archaea	106
Bacteria	1258

Table S1
