# Supplementary material for: The fundamental tradeoff in genomes and proteomes of prokaryotes established by the genetic code, codon entropy, and physics of nucleic acids and proteins
Source: Biol Direct. 2014 Dec 12;9:29. doi: 10.1186/s13062-014-0029-2 (PMC4273451; doi:10.1186/s13062-014-0029-2)
Supplement: Additional file 1: — Compilation of all supplementary figures and tables. Complete list of supplementary figures and tables is given in the file. [file 13062_2014_29_MOESM1_ESM.zip › 2012097662141560_add2.pdf]

Amino acid GC saturation scale																				
	**GC-poor group**					**GC-medium group**										**GC-rich group**				
**Amino acid**	**I**	**F**	**K**	**N**	**Y**	**M**	**L**	**C**	**D**	**E**	**H**	**Q**	**S**	**T**	**V**	**W**	**R**	**A**	**G**	**P**
**GC saturation**	0.11	0.17	0.17	0.17	0.17	0.33	0.39	0.5	0.5	0.5	0.5	0.5	0.5	0.5	0.5	0.67	0.72	0.83	0.83	0.83
**Total codons**	3	2	2	2	2	1	6	2	2	2	2	2	6	4	4	1	6	4	4	4
**GC-rich codons**	0-1	0-1	0-1	0-1	0-1	1	0-2	1-2	1-2	1-2	1-2	1-2	1-2	1-2	1-2	2	1-3	2-3	2-3	2-3
**Amino acid composition in all prokaryotic genomes**																				
**min**	2.24	1.97	1.09	1.37	1.60	1.15	7.98	0.18	2.28	3.30	0.97	1.19	3.40	2.64	2.07	0.02	1.86	1.48	3.70	1.62
**mean**	6.69	4.12	5.60	4.07	3.16	2.33	10.22	0.95	5.39	6.32	2.03	3.59	6.03	5.29	7.10	1.15	5.38	9.04	7.26	4.27
**median**	6.57	4.07	5.45	3.95	3.10	2.40	10.20	0.95	5.36	6.20	2.10	3.62	6.00	5.32	7.05	1.19	5.27	8.62	7.20	4.25
**max**	17.57	8.99	18.03	12.88	5.77	3.34	14.58	3.10	9.06	9.98	3.32	6.15	10.04	7.90	10.89	1.82	10.58	15.96	10.62	7.10
**stddev**	2.01	0.74	2.47	1.52	0.76	0.36	0.80	0.28	0.62	0.96	0.33	0.91	0.76	0.58	0.99	0.32	1.61	2.72	1.13	0.95

Table S2

Amino acid content prediction based on different regression models

	GC-poor group					GC-medium group										GC-rich group				
**Amino acid**	**I**	**F**	**K**	**N**	**Y**	**M**	**L**	**C**	**D**	**E**	**H**	**Q**	**S**	**T**	**V**	**W**	**R**	**A**	**G**	**P**
**GC saturation**	0.11	0.17	0.17	0.17	0.17	0.33	0.39	0.5	0.5	0.5	0.5	0.5	0.5	0.5	0.5	0.67	0.72	0.83	0.83	0.83
**Prediction based on protein-coding GC (individual weighted regression models for each amino acid)**																				
**RMSE**	0.64	0.41	0.85	0.53	0.39	0.36	0.76	0.28	0.59	0.89	0.29	0.91	0.61	0.56	0.68	0.2	0.57	0.85	0.39	0.36
**R^2**	0.9	0.69	0.88	0.88	0.73	0.02	0.11	0	0.1	0.14	0.24	0	0.35	0.06	0.53	0.61	0.87	0.9	0.88	0.86
**Prediction based on protein-coding GC (three regression models for the corresponding GC saturation groups)**																				
**RMSE**	0.63	0.42	0.84	0.52	0.4	0.36	0.8	0.28	0.62	0.96	0.33	0.9	0.76	0.58	0.99	0.21	0.57	0.85	0.39	0.36
**R^2**	0.9	0.68	0.89	0.88	0.73	0	0	0	0	0	0	0	0	0	0	0.58	0.87	0.9	0.88	0.86
**Prediction based on genomic GC (individual weighted regression models for each amino acid)**																				
**RMSE**	0.69	0.43	0.9	0.53	0.4	0.36	0.74	0.27	0.58	0.86	0.29	0.91	0.6	0.56	0.67	0.21	0.56	0.88	0.41	0.36
**R^2**	0.88	0.66	0.86	0.87	0.72	0.02	0.11	0	0.09	0.16	0.22	0	0.37	0.04	0.51	0.58	0.88	0.9	0.87	0.86
**Prediction based on genomic GC (three regression models for the corresponding GC saturation groups)**																				
**RMSE**	0.67	0.43	0.88	0.52	0.41	0.36	0.79	0.28	0.6	0.94	0.33	0.91	0.75	0.57	0.97	0.21	0.56	0.88	0.41	0.36
**R^2**	0.89	0.65	0.87	0.88	0.72	0	0	0	0	0	0	0	0	0	0	0.55	0.88	0.9	0.87	0.86

Table S3

	GC-poor group					GC-medium group										GC-rich group				
**Amino acid**	**I**	**F**	**K**	**N**	**Y**	**M**	**L**	**C**	**D**	**E**	**H**	**Q**	**S**	**T**	**V**	**W**	**R**	**A**	**G**	**P**
**GC saturation**	0.11	0.17	0.17	0.17	0.17	0.33	0.39	0.5	0.5	0.5	0.5	0.5	0.5	0.5	0.5	0.67	0.72	0.83	0.83	0.83
**Natural GC, [Predicted/actual GC_{NCB}]**	**GC_{NCB} and amino acid content prediction based on genomic GC**																			
	**Candidatus Zinderia insecticola CARI**																			
**13.2**	17.57	6.96	18.03	12.88	5.77	1.23	9.36	1.09	2.28	3.3	0.97	1.19	5.22	2.64	2.07	0.02	2.05	1.67	3.74	1.99
[30 / **30.30**]	14.97	6.93	15.82	10.32	6.02	2.5	9.09	1.03	4.48	7.76	1.24	3.16	7.81	4.43	3.86	0.04	-0.86	-1.9	2.78	0.52
	**Candidatus Carsonella ruddii PV**																			
**15.97**	15.52	8.99	14.8	11.78	5.57	1.22	10.15	1.63	2.62	3.33	1.05	1.48	6.34	3.18	3.22	0.46	1.86	1.48	3.7	1.62
[31.8 / **31.67**]	14.15	6.65	14.81	9.7	5.73	2.48	9.2	1.02	4.57	7.61	1.32	3.2	7.63	4.51	4.18	0.15	-0.24	-0.82	3.22	0.89
	**Streptococcus pneumoniae CGSP14**																			
**40.29**	7.24	4.67	6.95	4.4	3.85	2.42	10.24	0.62	5.48	7.22	1.94	4.07	6.58	5.49	6.85	0.94	4.1	7.3	6.4	3.23
[45.8 / **45.73**]	7.87	4.52	7.05	4.97	3.56	2.34	10.06	0.97	5.26	6.5	1.91	3.51	6.28	5.16	6.64	0.99	4.52	7.52	6.63	3.75
	**Frankia alni ACN14a**																			
**73.02**	3.22	2.55	1.22	1.5	1.75	1.33	10.13	0.77	6.17	4.76	2.19	2.52	5.11	5.96	8.78	1.42	8.91	14.75	10.03	6.92
[56.9 / **57.84**]	2.91	2.84	0.93	1.24	1.85	2.22	10.74	0.92	5.8	5.62	2.38	3.76	5.2	5.68	8.59	1.65	8.27	14.09	9.32	6
	**Cellulomonas flavigena DSM 20109**																			
**74.31**	2.32	2.32	1.22	1.37	1.76	1.29	10.28	0.6	6.65	5.1	2.23	2.63	4.58	6.56	10.89	1.6	8.24	14.7	9.39	6.26
[57.6 / **57.73**]	2.62	2.74	0.57	1.02	1.74	2.22	10.78	0.92	5.84	5.57	2.41	3.77	5.14	5.71	8.7	1.69	8.49	14.47	9.48	6.13

Tables S4

Name	tax_id	Start/ End	GC	NCB	CB	depth	H	R/Y	A	T	G	C	Trend, depth	Trend, R/Y
Kineococcus radiotolerans SRS30216 (B)	266940	S	0.745	0.5736	0.1714	0.9802	4.8482	0.9263	0.123	0.1319	0.3578	0.3872	down	up
		E	0.6882	0.6131	0.075	0.9052	5.6365	0.9487	0.1505	0.1613	0.3363	0.3518		
Polynucleobacter necessarius subsp. asymbioticus QLW-P1DMWA-1 (B)	312153	S	0.4526	0.4892	-0.0366	0.9868	5.6944	1.0334	0.2689	0.2785	0.2393	0.2133	same	same
		E	0.4808	0.4938	-0.013	0.9866	5.9061	0.9944	0.254	0.2652	0.2446	0.2362		
Methanoculleus marisnigri JR1 (A)	368407	S	0.6296	0.513	0.1166	0.989	5.4021	1.0449	0.1945	0.1759	0.3165	0.3131	down	same
		E	0.5991	0.5643	0.0347	0.9222	5.8476	1.016	0.2073	0.1936	0.2967	0.3024		
Candidatus Carsonella ruddii PV (B)	387662	S	0.1593	0.3166	-0.1573	1.0373	4.5304	1.1855	0.45	0.3907	0.0924	0.0669	up	down
		E	0.2428	0.3478	-0.105	1.0898	5.2371	1.0407	0.3757	0.3815	0.1343	0.1086		
Streptobacillus moniliformis DSM 12112 (B)	519441	S	0.262	0.4068	-0.1448	0.9772	4.991	1.435	0.4202	0.3178	0.1691	0.0928	up	down
		E	0.3414	0.4135	-0.0722	1.0235	5.6032	1.2311	0.3442	0.3145	0.2076	0.1337		
Lactobacillus delbrueckii subsp. bulgaricus ND02 (B)	767455	S	0.5125	0.4676	0.0449	0.9705	5.5686	1.1036	0.2641	0.2234	0.2605	0.252	down	down
		E	0.5211	0.5175	0.0037	0.9485	5.9108	1.0421	0.2518	0.2271	0.2586	0.2626		
**Simulation of two genomes with distorted codon bias**														
Streptobacillus moniliformis DSM 12112 (B)	519441	S	0.5525	0.4068	0.1457	0.9772	4.9623	1.2117	0.2704	0.1771	0.2775	0.275	down	down
		E	0.5506	0.5359	0.0147	0.9116	5.881	1.1274	0.2585	0.1909	0.2714	0.2792		
Nocardiopsis dassonvillei subsp. Dassonvillei DSM 43111(B)	446468	S	0.4227	0.5654	-0.1427	0.9687	5.133	1.1841	0.2883	0.289	0.2539	0.1688	up	down
		E	0.4572	0.4884	-0.0313	0.991	5.8629	1.1161	0.2683	0.2745	0.2591	0.1981		

Tables S5

Examles of amino acid content prediction in genomes not included in the original dataset.

	GC-poor group					GC-medium group										GC-rich group				
**Amino acid**	**I**	**F**	**K**	**N**	**Y**	**M**	**L**	**C**	**D**	**E**	**H**	**Q**	**S**	**T**	**V**	**W**	**R**	**A**	**G**	**P**
**GC saturation**	0.11	0.17	0.17	0.17	0.17	0.33	0.39	0.5	0.5	0.5	0.5	0.5	0.5	0.5	0.5	0.67	0.72	0.83	0.83	0.83
**GC_NCB**	**Borrelia crocidurae str. Achema** GC genomic = **29.06,** GC coding = **27.79**																			
**Natural: 40.06**	11.03	5.99	9.53	7.34	4.37	1.93	10.24	0.74	5.5	6.54	1.43	2.59	7.01	4.24	5.56	0.52	3.4	4.37	5.21	2.45
**Predicted: 41.23**	9.79	5.27	9.41	6.42	4.34	2.33	10.22	0.95	5.39	6.32	2.03	3.59	6.03	5.29	7.1	0.66	2.92	4.89	5.53	2.82
**GC_NCB**	**Halobacillus halophilus DSM 2266** GC genomic = **41.80,** GC coding = **42.41**																			
**Natural:46.45**	7.17	4.49	6.34	4.18	3.58	2.83	9.61	0.63	5.29	8.02	2.29	4	6.33	5.32	6.98	1.11	4.27	6.85	6.97	3.75
**Predicted: 46.37**	7.59	4.45	6.7	4.75	3.5	2.33	10.22	0.95	5.39	6.32	2.03	3.59	6.03	5.29	7.1	1.01	4.67	7.84	6.76	3.85
**GC_NCB**	**Rubrivivax gelatinosus IL144** GC genomic = **71.2,** GC coding = **71.47**																			
**Natural: 55.44**	3.53	3.23	2.43	2.03	1.98	2	11.17	0.89	5.32	5.59	2.16	3.49	4.78	4.87	7.95	1.55	8.17	14.68	8.59	5.57
**Predicted: 56.09**	3.42	2.91	1.58	1.61	1.93	2.33	10.22	0.95	5.39	6.32	2.03	3.59	6.03	5.29	7.1	1.66	7.97	13.41	9.07	5.81
**GC_NCB**	**Phycisphaera mikurensis** GC genomic = **73.23,** GC coding = **73.19**																			
**Natural: 56.73**	3.04	3.26	2.14	1.94	1.71	1.66	10.48	0.9	6.05	6	2.15	2.42	4.71	4.9	7.81	1.4	8.33	14.81	9.89	6.41
**Predicted: 57.04**	3.01	2.76	1.08	1.3	1.77	2.33	10.22	0.95	5.39	6.32	2.03	3.59	6.03	5.29	7.1	1.73	8.3	13.95	9.3	6

Table S6

Genome	Protein-coding GC	GC_{NCB}	GC_{CB}
(red) Campylobacter curvus 525.92	45.28	44.88	0.48
(red) Polynucleobacter necessarius subsp. asymbioticus QLW-P1DMWA-1	45.26	49.10	-3.84
(green) Lactobacillus delbrueckii subsp. bulgaricus ND02	51.25	46.94	4.31
(green) Prochlorococcus marinus str. MIT 9303	51.25	51.15	0.1
(magenta) Methanoculleus marisnigri JR1	62.96	51.53	11.43
(magenta) Isosphaera pallida ATCC 43644	62.99	54.20	8.79

Table S7

Group of genomes	Parameters of the logistic function if fitted to a group of genomes instead of the whole data set			
	**a**	**b**	**c**	**r**
Aerobes	17.87	-11.6	50.16	0.37
Anaerobes	22.01	-27.25	46.30	0.18
Host-associated	18.02	-14.12	49.48	0.31
Terrestrial	22.05	-30.24	46.15	0.19
Mesophiles	19.17	-15.75	49.16	0.29
Hyperthermophiles	6.077	-12.40	45.74	0.67
Bacteria	20.06	-15.30	49.54	0.28
Archaea	20.19	-28.26	45.58	0.19

Table S8
